# pH-responsive DNA nanomicelles for chemo-gene synergetic therapy of anaplastic large cell lymphoma

**DOI:** 10.7150/thno.45803

**Published:** 2020-07-09

**Authors:** Yuwei Li, Shuzhen Yue, Jingyu Cao, Chengzhan Zhu, Yixiu Wang, Xin Hai, Weiling Song, Sai Bi

**Affiliations:** 1 Research Center for Intelligent and Wearable Technology, College of Chemistry and Chemical Engineering, Qingdao University, Qingdao 266071, P. R. China.; 2 Laboratory of Optic-electric Sensing and Analytical Chemistry for Life Science, MOE, Shandong Key Laboratory of Biochemical Analysis, Key Laboratory of Analytical Chemistry for Life Science in Universities of Shandong, College of Chemistry and Molecular Engineering, Qingdao University of Science and Technology, Qingdao 266042, P. R. China.; 3 Department of Hepatobiliary and Pancreatic Surgery, Affiliated Hospital of Qingdao University, Qingdao 266003, P. R. China.

**Keywords:** DNA nanomicelles, pH stimuli, chemotherapy, gene therapy, anaplastic large cell lymphoma

## Abstract

Chemo-gene therapy is an emerging synergetic modality for the treatment of cancers. Herein, we developed pH-responsive multifunctional DNA nanomicelles (DNMs) as delivery vehicles for controllable release of doxorubicin (Dox) and anaplastic lymphoma kinase (ALK)-specific siRNA for the chemo-gene synergetic therapy of anaplastic large cell lymphoma (ALCL).

**Methods:** DNMs were synthesized by performing in situ rolling circle amplification (RCA) on the amphiphilic primer-polylactide (PLA) micelles, followed by functionalization of pH-responsive triplex DNA via complementary base pairing. The anticancer drug Dox and ALK-specific siRNA were co-loaded to construct Dox/siRNA/DNMs for chemo-gene synergetic cancer therapy. When exposed to the acidic microenvironment (pH below 5.0), C-G·C^+^ triplex structures were formed, leading to the release of Dox and siRNA for gene silencing to enhance the chemosensitivity in ALCL K299 cells. The chemo-gene synergetic anticancer effect of Dox/siRNA/DNMs on ALCL was evaluated *in vitro* and *in vivo*.

**Results:** The pH-responsive DNMs exhibited good monodispersity at different pH values, good biocompatibility, high drug loading capacity, and excellent stability even in the human serum. With the simultaneous release of anticancer drug Dox and ALK-specific siRNA in response to pH in the tumor microenvironment, the Dox/siRNA/DNMs demonstrated significantly higher treatment efficacy for ALCL compared with chemotherapy alone, because the silencing of ALK gene expression mediated by siRNA increased the chemosensitivity of ALCL cells. From the pathological analysis of tumor tissue, the Dox/siRNA/DNMs exhibited the superiority in inhibiting tumor growth, low toxic side effects and good biosafety.

**Conclusion:** DNMs co-loaded with Dox and ALK-specific siRNA exhibited significantly enhanced apoptosis of ALCL K299 cells *in vitro* and effectively inhibited tumor growth *in vivo* without obvious toxicity, providing a potential strategy in the development of nanomedicines for synergetic cancer therapy.

## Introduction

Anaplastic large cell lymphoma (ALCL) is an aggressive systemic disease, which occurs most commonly in children and young adults. The expression of anaplastic lymphoma kinase (ALK) oncogene is a critical factor in the development of lymphoma 1. Chemotherapy is one of the most common methods of cancer therapy 2,3. However, the therapeutic effect is seriously impeded due to the adverse side effects and inefficient delivery of anticancer drugs. Furthermore, a single treatment modality against ALCL is often ineffective against the heterogeneity of tumor tissues, and may induce drug-resistance in lymphoma cells. It has been reported that siRNA-mediated knockdown of ALK gene can downregulate the expression of the nucleophosmin-anaplastic lymphoma kinase (NPM-ALK) 4. NPM-ALK plays an important role in the regulation of cancer cell growth. The ALK-specific siRNA resulted in the specific reduction of ALK gene expression in ALCL cells and interference with the fusion protein function, which could be exploited in cancer therapy 4-7. More recently, combination therapy has gained increasing research interest for its cumulative effects in cancer treatment 8. Notably, some studies have reported that the combination of ALK-specific siRNA with chemotherapeutic agents, such as doxorubicin (Dox), can augment the chemosensitivity 6,7. Thus, the development of efficient combination therapeutic delivery vehicles for ALCL is urgently needed.

As an endogenous regulatory pathway initiated by siRNAs or microRNAs, RNA interference (RNAi) has become a promising tool in suppressing the expression of specific genes related to many human diseases, such as cancers 9 and liver cirrhosis 10. For gene knockdown, siRNA is incorporated into the RNA-induced silencing complex (RISC) to degrade the target mRNA, suppressing the expression of specific target genes 11. However, the effectual encapsulation and delivery of siRNAs for this therapeutic strategy is still a challenge 12. Besides, the cell penetration efficiency of the negatively charged siRNAs is limited because of the inability to cross the cell membrane. Several nanomaterial-based therapeutic platforms have been developed to address these problems, but they still suffer from cytotoxicity and inflammatory response 13,14. Alternatively, based on the highly specific Watson-Crick base pairing (A-T and G-C), DNA nanotechnology has emerged as a promising tool to construct functional DNA nanostructures with excellent biocompatibility, and has been widely applied in the fields of biological detection 15,16, biophysical studies 17 and nanomedicine 18-21.

In recent years, the DNA nanotechnology-based stimuli-responsive drug delivery systems have been constructed for controllable release of therapeutic agents, in which the conformational switches of DNAs could be triggered by factors, such as pH 22-25, temperature 26,27, and redox species 28,29. pH plays an essential role in diverse physiological processes, such as enzyme catalysis, secretory function, and protein folding 30. Abnormal intracellular pH is related to many diseases, such as cancers and Alzheimer's disease 31. Several pH-responsive DNA molecular probes have been developed to monitor the pH gradient 32-35. For example, the DNA triplex-based pH-responsive DNA switches were assembled by both Watson-Crick interactions and Hoogsteen or reverse Hoogsteen interactions 36-38. In these systems, the cytosine could be protonated under acidic conditions, subsequently binding to C-G to form the C-G·C^+^ triple DNA structure, whereas the structure reconfigured into the C-G duplex configuration at the neutral or basic pH.

Rolling circle amplification (RCA) is an isothermal enzymatic process that generates long, single-stranded DNA (ssDNA) from a circular DNA template in the presence of DNA polymerase, a short DNA primer and deoxynucleotide triphosphates (dNTPs) 39. Due to their programmability and high efficiency, RCA-based strategies have been extensively used in biosensing 40, bioimaging 41,42 and biomedicine 43,44 with the advantages of signal amplification, high stability, low cost, and easy functionalization. Herein, we propose pH-responsive DNA nanomicelles (DNMs) based on RCA to co-deliver anticancer drug doxorubicin (Dox) and ALK-specific siRNA for combined chemotherapy and gene therapy of ALCL. The RCA reaction occurred on the surface of primer-PLA micelles to produce ssDNA concatemers with multiple tandem repeats, facilitating the periodic assembly of numerous pH-responsive DNA probes and efficient intercalation with Dox. Both *in vitro* and *in vivo* studies demonstrated the efficient gene knockdown and cancer therapeutic abilities of the proposed DNMs, which hold great potential in nanomedicine and cancer theranostics.

## Results and Discussion

### RCA-based, pH-responsive DNMs for Chemo-Gene Synergetic Therapy

**Scheme 1:**
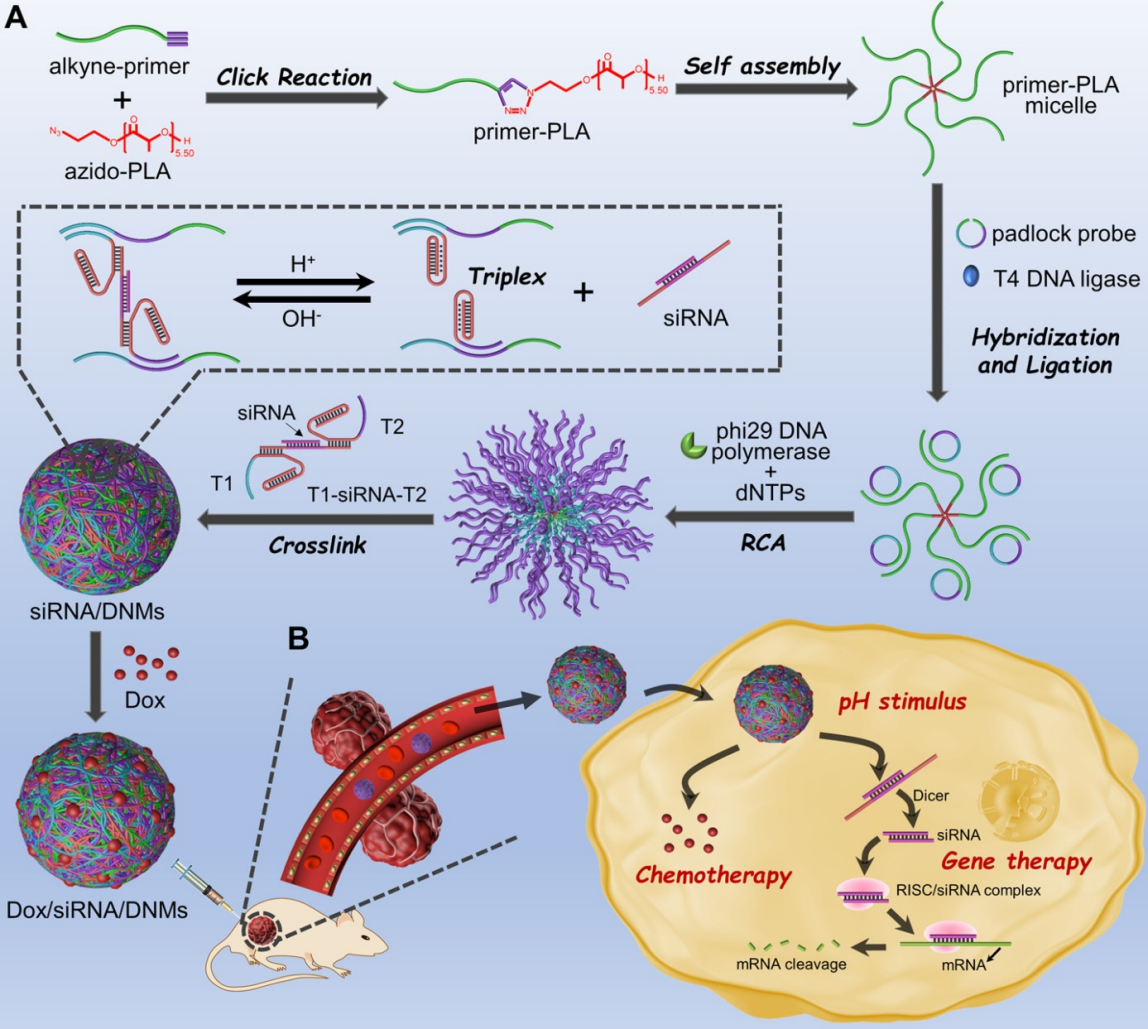
Schematic of pH-responsive DNMs co-loaded with anticancer drug Dox and ALK-specific siRNA for synergetic chemo-gene therapy of ALCL. (**A**) Construction and (**B**) delivery of Dox/siRNA/DNMs to K299 cells for synergetic chemo-gene therapy.

The principle of RCA-based pH-responsive DNMs co-loaded with Dox and ALK-specific siRNA for synergistic anticancer therapy is displayed in **Scheme 1**. First, PLA is functionalized with azide via ring-opening polymerization of DL-lactide. The DNA primer-PLA conjugate is synthesized using the azide-modified PLA and alkyne-modified DNA primer through the click reaction of efficient copper (I)-catalyzed alkyne-azide cycloaddition (CuAAC) to form the amphiphilic DNA-polymer micelles. Next, the circular template DNA is prepared by ligating the padlock probe on the primer with a T4 DNA ligase to perform the RCA reaction. The circular template DNA includes three regions: the green domain depicts the primer-binding site, and the blue and purple domains via RCA generate the complementary sequences of triplex DNA 1 and DNA 2 (T1 and T2), respectively. The RCA process is initiated on the primer-PLA micelles by adding phi29 DNA polymerase and dNTPs to generate a large number of long ssDNAs with repetitive sequences that are complementary to the circular template. The resulting RCA products are further cross-linked with the T1-ALK-specific siRNA-T2 probes via Watson-Crick base-pairing, to achieve the assembly of stable DNMs at weak acidic/neutral/basic pH. Under the acidic tumor microenvironment, the T1 and T2 form the closed triplex structures and stay in the off state, resulting in the release of ALK-specific siRNA via pH stimuli.

The base composition and secondary structure of T1-siRNA-T2 hybrid in response to pH are presented in **Figure S1**. The RCA-based DNMs provide numerous drug loading sites for anticancer drugs, such as Dox, which can be released under acidic conditions (**Scheme 1A**). Because of the pH-responsive mechanism dependent on the Hoogsteen interactions for triplex-helix molecular switch 33,36, the DNMs undergo a conformational change responsive to the acidic environment in endosomes and lysosomes when Dox/siRNA/DNMs are transported into cancer cells via endocytosis, leading to the release of siRNA 45. Subsequently, the therapeutic drugs can escape to the cytoplasm via the “proton sponge effect” 46,47, achieving combination cancer therapy (**Scheme 1B**). In summary, by using the triplex DNA for siRNA loading and pH-stimulated release of siRNA together with the chemotherapeutic drug Dox, the developed DNM-based delivery system with excellent biostability has a great potential for the chemo-gene synergetic therapy of cancers.

Compared to other biopolymers, DNA-based micelles have advantages of nucleotide programmability and controllable supramolecular structure 48. Furthermore, DNA micelles have good biocompatibility and high stability both *in vitro* and* in vivo* 49 and have, therefore, become an efficient tool in biomedical research 50-52. In recent years, RCA-based strategies have been widely applied in biomedicine, in which the assembled products function as carriers for the delivery of therapeutic oligonucleotides, proteins and drugs 21,53,54. However, the co-delivery of multiple types of therapeutic drugs is still a challenge in RCA-based biomedical applications.

In this study, nucleic acids were conjugated with hydrophobic polymers to construct three-dimensional micelle nanostructures. We designed the amphiphilic primer-PLA micelles to perform the RCA reaction in situ, resulting in the generation of long ssDNA products on micelles with condensed nanostructures to co-load chemo-drugs and siRNAs for chemo-gene synergetic therapy. Notably, a single primer-PLA micelle could simultaneously perform multiple RCA reactions, increasing the local concentration of RCA products. Thus, the T1-siRNA-T2 hybrids were likely to hybridize with the DNA micelles, which helped accelerate the hybridization kinetics and increase the loading of siRNA. More importantly, the proposed Dox/siRNA/DNMs were responsive to acidic pH based on the triplex-helix molecular switch, achieving the precise control of drug release in the tumor microenvironment.

### Characterization of DNMs

**Figure 1:**
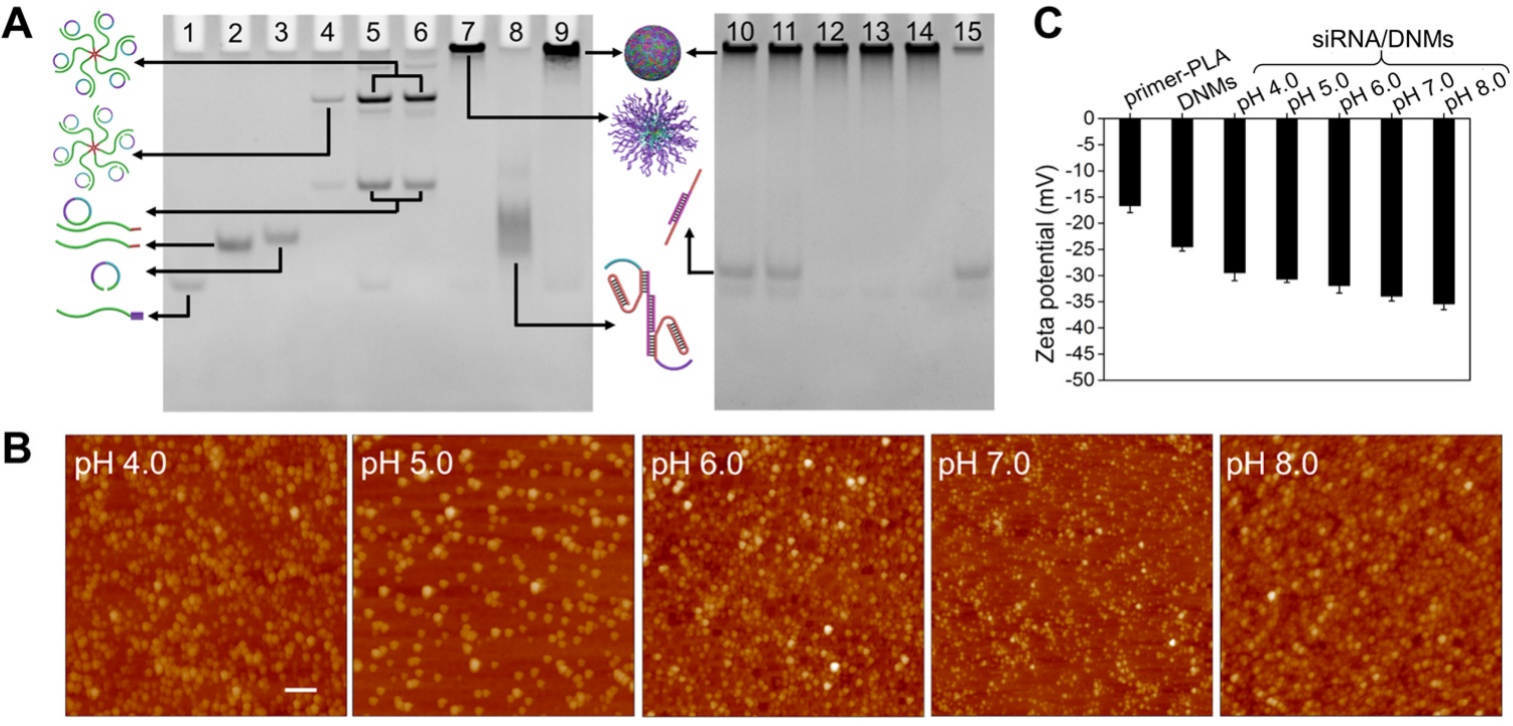
** Characterization of DNMs.** (**A**) Characterization of the assembly pathways of DNMs and the response to pH stimulation by 8% native PAGE. Lane 1: primer; lane 2: primer-PLA; lane 3: padlock probe; lane 4: annealed product of padlock probe and primer-PLA micelle; lane 5: products of lane 4 treated with T4 DNA ligase; lane 6: products of lane 5 treated with Exo I; lane 7: RCA products on primer-PLA micelles; lane 8: hybrids of T1, siRNA, and T2 (T1-siRNA-T2); lane 9: T1-siRNA-T2-loaded DNMs (siRNA/DNMs). Lanes 10-14: siRNA/DNMs at pHs from 4.0 to 8.0; lane 15: siRNA. (**B**) AFM images of siRNA/DNMs at pHs from 4.0 to 8.0. Scale bar: 1 μm. (C) Zeta potentials of primer-PLA, DNMs and siRNA/DNMs at different pHs. Error bars indicate SD (n = 3).

The primer-PLA conjugate was synthesized using the alkyne-modified DNA primer and azide-modified PLA through efficient CuAAC. To functionalize PLA with an azide group, 2-azido-ethanol was used as an initiator and 1, 8-diazabicyclo[5.4.0]undec-7-ene (DBU) served as an organocatalyst to achieve the controlled ring-opening polymerization of DL-lactide. The ^1^H NMR and FTIR characterization of the synthesized azido-PLA are shown in **Figure S2**. The assembly pathways of siRNA-loaded DNMs (siRNA/DNMs) were verified by polyacrylamide gel electrophoresis (PAGE) (**Figure 1A**). Compared with the free primer (lane 1), the primer-PLA composite showed slow mobility (lane 2), indicating the successful conjugation of alkyne-modified DNA primer and azide-modified PLA via CuAAC reaction. The critical micelle concentration (CMC) of DNA-PLGA nanomicells was reported to be ~ 7.5 mg/L 55. Since the CMC increased with the decrease of the hydrophobic carbon chain, the CMC of primer-PLA nanomicells was likely > 7.5 mg/L. In PAGE experiment, the final 0.2 μM (equal to 0.37 mg/L) concentration of primer-PLA in lane 2 was below the CMC. Thus, at this concentration, the primer-PLA could not assemble into micelles 2,56. For the RCA reaction, the annealed product of the padlock probe and primer-PLA micelle yielded reduced mobility (lane 4). There was no obvious change after the padlock probe was circularized with T4 DNA ligase (lane 5) and further treated with exonuclease I (Exo I) to remove the excess primers (lane 6). The other bands in lanes 4, 5, and 6 might be the hybrid products of the padlock probe and primer-PLA after annealing, ligation, and treatment with Exo I, respectively. Since excess primers were used in the circular template preparation, a band corresponding to the primers was observed in lane 5. Addition of phi29 DNA polymerase and dNTPs to initiate RCA on the primer-PLA micelles resulted in a bright band with slow gel mobility (lane 7). Subsequently, the hybrids of T1-siRNA-T2 were formed at pH 7.4 (lane 8), which hybridized with RCA products to produce a brighter band than that in lane 7 at the top of the gel (lane 9). The reaction temperature and reaction time of RCA were optimized as 30 °C for 3 h (**Figure S3**).

The siRNA/DNMs were subjected to pH values ranging from 4.0 to 8.0 to verify the pH response of the proposed DNMs based on the formation of triplex DNA under acidic conditions and characterized by native PAGE (**Figure 1A**), atomic force microscopy (AFM) (**Figure 1B**), and zeta potential measurement (**Figure 1C**). As shown in native PAGE (**Figure 1A**), bands of siRNA appeared at pH 4.0 and 5.0 (lanes 10 and 11), demonstrating the release of siRNA under acidic conditions due to the formation of C-G·C^+^ bridges. In contrast, no siRNA band was observed when pH increased from 6.0 to 8.0 (lanes 12 to 14) because of the stable interaction of T1-siRNA-T2 with RCA products via Watson-Crick base pairing.

From the AFM characterization (**Figure 1B**), the assembled DNMs demonstrated good monodispersity at different pH values. When the pH increased from 4.0 to 8.0, the sizes of DNMs reduced from 281.88 to 135.08 nm, which could be attributed to the condensation of RCA products via cross-linking with T1-siRNA-T2 hybrids. Scanning electron microscopy (SEM) was also carried out to characterize the morphologies of DNMs and siRNA/DNMs at pH 7.0, both of which demonstrated three-dimensional spherical structures (**Figure S4**).

Dynamic light scattering (DLS) was used to determine the average sizes of siRNA/DNMs at different pH values (**Figure S5**). The results showed a reduction in the average sizes of siRNA/DNMs from 298.3 to 166.7 nm when the pH increased from 4.0 to 8.0, consistent with the results obtained from AFM characterization. The zeta potentials of the primer-PLA, DNMs, and siRNA/DNMs were also measured at different pH values to further confirm the assembly steps (**Figure 1C**). Compared with primer-PLA, the more negative zeta potential of DNMs indicated the RCA was performed in situ on the primer-PLA micelles. Furthermore, the zeta potentials of the siRNA/DNMs assembled at pH 6.0 - 8.0 were more negative than those at pH 4.0 and 5.0, suggesting that more T1-siRNA-T2 units were incorporated into the DNMs at weak acidic/neutral/basic pH, and were more stable than those at strong acidic pH.

### Drug Loading Capacity and Stability of Dox/siRNA/DNMs

**Figure 2:**
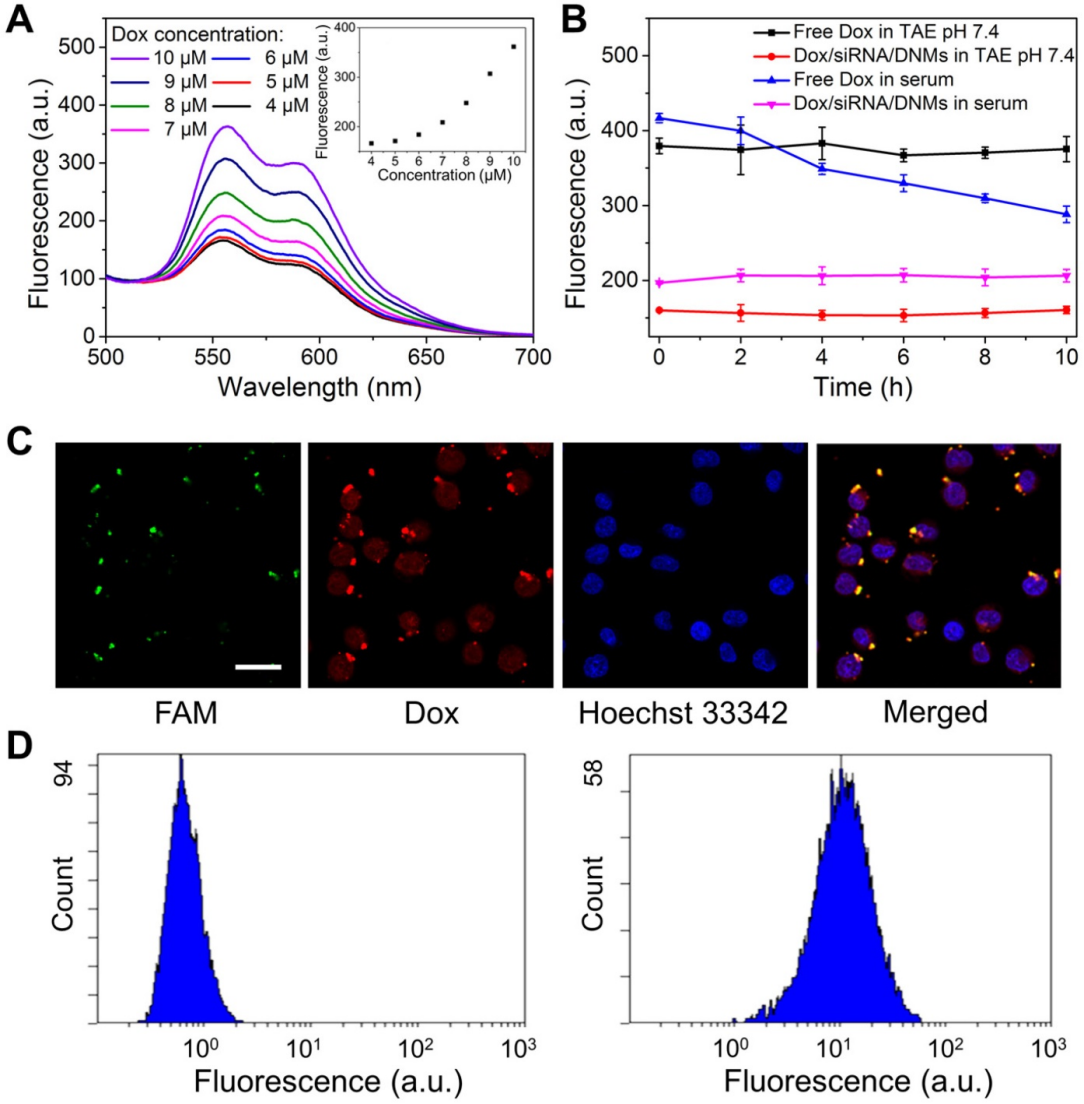
Drug loading capacity and intercellular behavior of DNM-based nanocarriers. (**A**) Fluorescence spectra of different amounts of Dox incubated with siRNA/DNMs in TAE (pH 7.4). Insert: Fluorescence intensity of Dox/siRNA/DNMs at the emission wavelength of 555 nm. (**B**) Stability of Dox/siRNA/DNMs incubated in TAE (pH 7.4) and normal human serum. Error bars indicate SD (n = 3). (**C**) CLSM imaging of the distribution of FAM-labeled Dox/siRNA/DNMs in K299 cells. The nucleus is stained with Hoechst 33342 (blue). The transfection time is 8 h. Scale bar: 50 µm. (**D**) Flow cytometry results of K299 cells treated with 1× TAE (left) and FAM-labeled Dox/siRNA/DNMs (right).

It is well established that the anticancer drug Dox can be intercalated into the double-stranded 5'-GC-3' or 5'-CG-3' because of the presence of flat aromatic rings, leading to the fluorescence quenching of Dox 57,58. Furthermore, Dox can also interact with siRNAs via noncovalent intercalation and electrostatic interactions 59. In the pH-responsive siRNA/DNMs, the double-stranded domains produced by the hybridization of RCA products with T1-siRNA-T2 hybrids could provide sufficient drug loading sites for Dox. Different concentrations of Dox were intercalated into a fixed amount of siRNA/DNMs to determine the drug payload capacity of the siRNA/DNMs (**Figure 2A**). As the Dox concentration decreased from 10 to 5 μM, the fluorescence intensities gradually decreased. Notably, when Dox concentration was below 5 μM, no noticeable fluorescence change was observed, indicating the maximum or saturated drug loading amount of siRNA/DNMs to be 5 μM. Thus, the final concentration of 5 μM Dox was chosen to prepare the Dox/siRNA/DNMs. The loading efficiency and capacity of siRNA and Dox in DNMs were further investigated in detail (see Supporting Information).

Besides high drug loading capacity, an ideal drug-delivery system should be stable under physiological conditions 60. To confirm this, we evaluated the stability of Dox/siRNA/DNMs in TAE buffer and human serum (**Figure 2B** and **Figure S7**). At different time intervals, fluorescence measurements showed that almost no Dox was released not only in TAE buffer (pH 7.4) but also in human serum. It has been reported that the pH value of serum increased with time 61 and the fluorescence intensity of free Dox decreased with the increase in pH values 62. The decreased fluorescence intensity of free Dox with time was attributed to the pH change of serum 60. Also, the loaded siRNA could be protected by the DNMs from degradation in complex physiological environment. Thus, the siRNAs in DNMs were stable in the extracellular environment and could be successfully delivered into cells. The excellent biostability of the Dox/siRNA/DNMs affords their wide application in biomedical research.

Given the high drug loading capacity and excellent stability of Dox/siRNA/DNMs, we investigated their performance in Karpas 299 cells (K299 cells). The DNMs were visualized by labeling T1 and T2 with FAM. The green and red fluorescence imaged by confocal laser scanning microscopy (CLSM) corresponded to the fluorescence of FAM and Dox, respectively, indicating the successful transport of Dox/siRNA/DNMs into K299 cells within 8 h (**Figure 2C**). The K299 cells were also stained with Hoechst 33342 to identify cell nuclei. The merged yellow color observed by CLSM indicated the overlap of green fluorescence from FAM-labeled T1 and T2 with the red fluorescence from Dox. The siRNA and Dox release after the Dox/siRNA/DNMs internalization into the cells allowed gene silencing and enhanced chemotherapeutic efficacy, resulting in K299 cell apoptosis. These results were also confirmed by flow cytometry analysis (**Figure 2D**). An obvious shift of fluorescence signal was observed in FAM-labeled DNMs-treated K299 cells compared with the control group. These results demonstrated that DNMs can serve as efficient vehicles for simultaneous encapsulation and delivery of gene therapy agents (siRNA) and chemotherapy drugs (Dox) to enhance the anticancer ability.

### Knockdown Efficiency of DNM-based Nanocarriers

**Figure 3:**
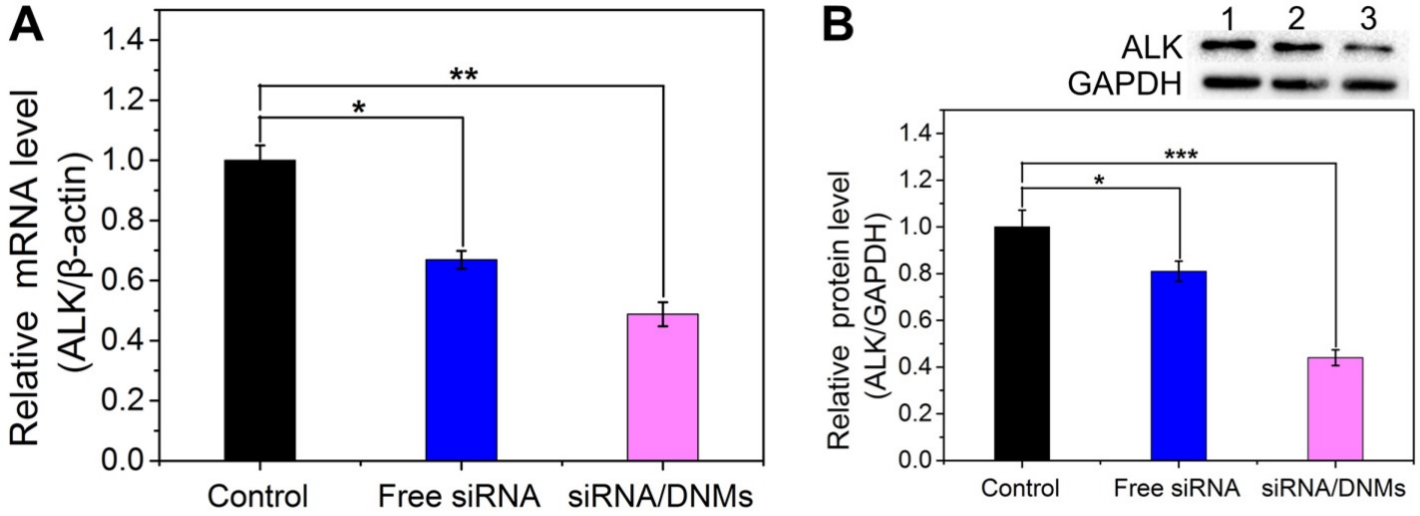
mRNA (**A**) and protein (**B**) expression levels of the ALK gene in K299 cells treated with 1× TAE (control), free siRNA and siRNA/DNMs. The qRT-PCR and western blotting analysis for the evaluation of mRNA silencing carried out by treating the K299 cells for 48 h. Error bars indicate SD (n = 3). *p ˂ 0.05, **p ˂ 0.01, and ***p ˂ 0.001.

The knockdown efficiency of siRNA/DNMs was determined by relative mRNA and protein expression levels using quantitative real-time polymerase chain reaction (qRT-PCR) and western blotting analysis. As shown in **Figure 3A**, the free ALK-specific siRNA exhibited 33.1% down-regulation at the mRNA level, while the siRNA/DNMs efficiently inhibited the ALK mRNA transcription by 51.2%. As displayed in **Figure 3B**, the free siRNA had a slight inhibitory effect on the ALK expression (19.0% reduction) compared to the control group. In contrast, the siRNA/DNMs-treated group achieved a significant gene knockdown efficiency of 56.0% at the protein expression level. The knockdown efficiency of DNMs carrying the negative control siRNA (NC siRNA/DNMs) evaluated by PCR and western blotting showed little ALK gene effect (**Figure S8**). Thus, the data revealed an excellent performance of ALK-specific siRNA/DNMs in gene silencing.

### Anticancer Efficacy of Dox/siRNA/DNMs *In Vitro*

**Figure 4:**
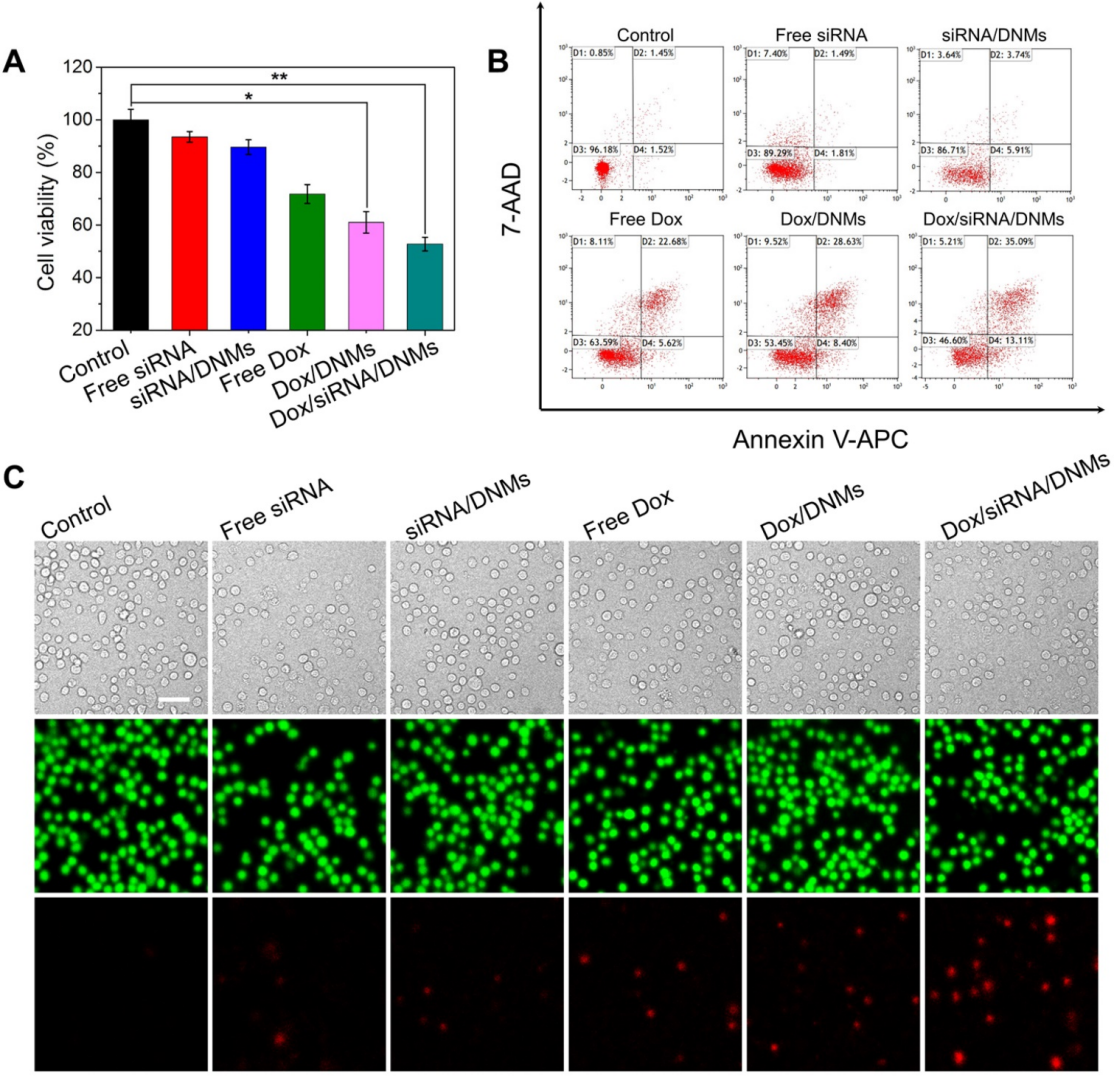
Anticancer efficacy of Dox/siRNA/DNMs *in vitro*. (**A**) CCK-8 assay, (**B**) flow cytometry analysis and (**C**) CLSM imaging of K299 cells after different treatments, including K299 cells treated with 1× TAE, free siRNA, siRNA/DNMs, free Dox, Dox/DNMs, and Dox/siRNA/DNMs. The flow cytometry results were obtained by double staining the K299 cells with Annexin V-APC/7-AAD, and the CLSM images were obtained by co-staining K299 cells with Calcein-AM (green) and PI (red). Error bars indicate SD (n = 3). *p ˂ 0.05, **p ˂ 0.01, and ***p ˂ 0.001. Scale bar: 200 µm.

To investigate the synergistic antitumor efficacy of the DNM-based nanocarriers, the cytotoxicities of free siRNA, siRNA/DNMs, free Dox, Dox/DNMs, and Dox/siRNA/DNMs on K299 cells were evaluated using the cell counting kit-8 (CCK-8) assay. K299 cells were treated with different formulations for 24 h. As shown in **Figure 4A**, free siRNA, siRNA/DNMs, free Dox, and Dox/DNMs exhibited 93.52%, 89.59%, 71.80% and 61.01% cell viability, respectively. The limited anticancer effect of siRNA/DNMs might be attributed to the relatively long half-life of ALK fusion proteins, in which the residual amount of NPM-ALK provides enough proliferative survival signals 4,6. Notably, inhibition of NPM-ALK could increase the chemosensitivity of tumor cells to Dox 5. Compared with siRNA/DNMs and Dox/DNMs, K299 cells incubated with Dox/siRNA/DNMs exhibited the lowest cell viability (52.75%), indicating the synergistic effect of the co-delivery of ALK-specific siRNA and Dox in enhancing tumor cell apoptosis 43,44. The combination index (CI) was also calculated to confirm the synergistic therapeutic effect of Dox/siRNA/DNMs (see Supporting Information for detail).

To further validate the enhanced therapeutic effect of Dox/siRNA/DNMs, apoptosis rates of K299 cells treated with different formulations were investigated by flow cytometry (**Figure 4B**). In each treatment, the final concentration of the loaded Dox was 100 nM. K299 cells were double stained with Annexin V-APC/7-AAD for apoptosis analysis. In the four-quadrant diagram, D1 and D3 represent the double positive and double negative cells of Annexin V-APC and 7-AAD fluorescence, refering to viable cells, respectively. D2 and D4 represent single positive cells of Annexin V-APC and 7-AAD fluorescence, referring to late apoptotic or necrotic cells and the early apoptotic cells, respectively. As expected, K299 cells treated with Dox/siRNA/DNMs demonstrated the highest apoptotic rate (48.16%). The results were further confirmed using Calcein-AM/PI double stain kit (**Figure 4C**). This method could identify the viable cells stained by Calcein-AM in green and the dead cells stained by PI in red simultaneously. From the CLSM images, the staining results were consistent with those obtained by flow cytometry. Thus, the DNMs for efficient co-delivery of the anticancer drug and therapeutic gene rapidly released Dox and siRNA triggered by the intracellular acidic environment, demonstrating an enhanced therapeutic efficiency and providing a potential approach for chemo-gene synergetic therapy of cancers.

### Evaluation of *In Vivo* Therapeutic Efficacy

**Figure 5:**
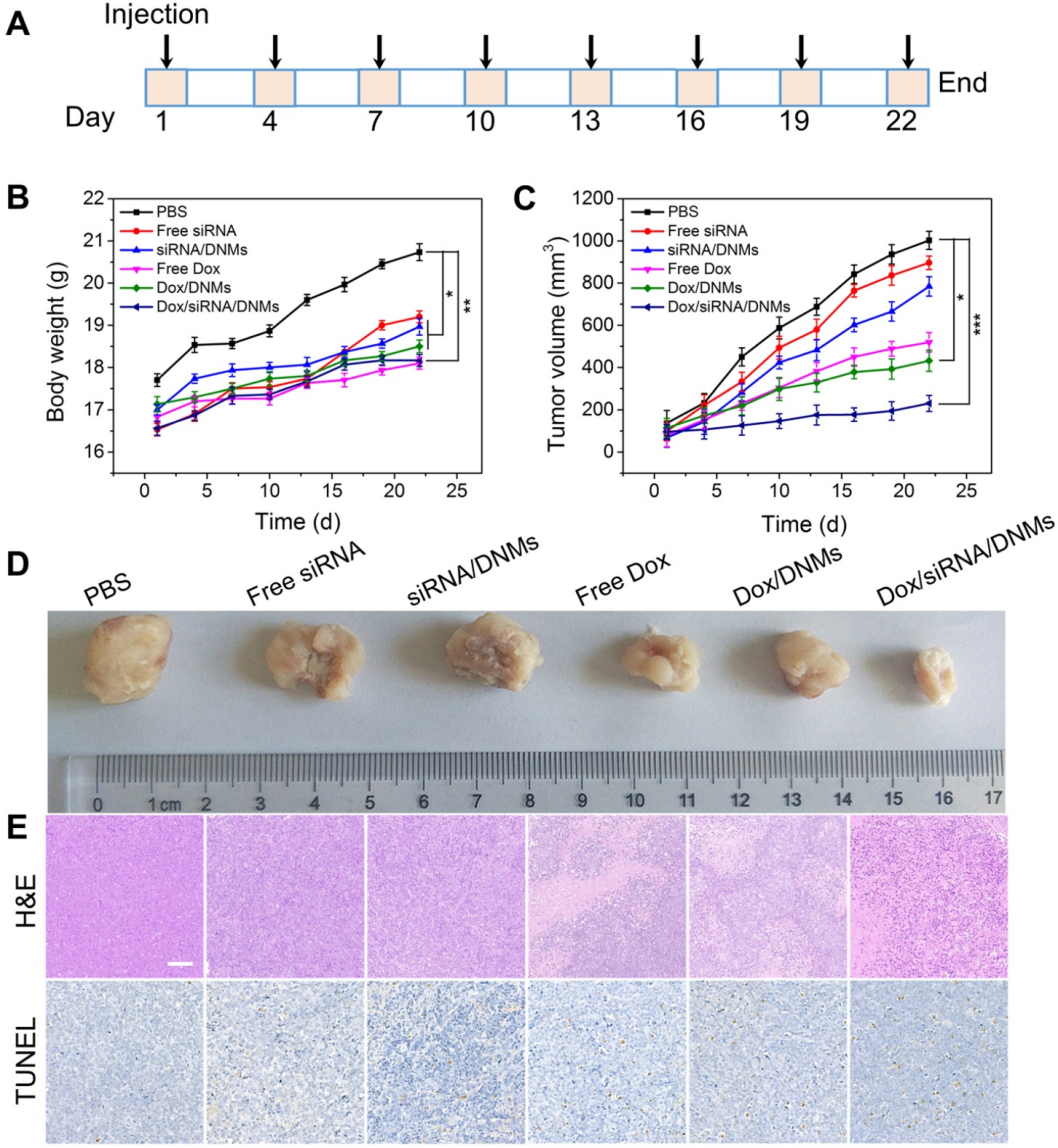
*In vivo* therapeutic study. (**A**) Time schedule of the treatment process. (**B**) Bodyweight changes of mice in different groups during therapy. (**C**) Tumor volume changes of different groups during therapy. Error bars indicate SD (n = 3). (**D**) Photographs of tumors dissected from the K299 tumor-bearing mice at the therapeutic terminal. (**E**) H&E and TUNEL staining of the tumor tissues treated with different groups. Scale bar: 100 µm. *p ˂ 0.05, **p ˂ 0.01, and ***p ˂ 0.001 (n = 3).

The pharmacokinetics and biodistribution studies demonstrated that Dox/siRNA/DNMs maintained a high concentration of Dox in systemic circulation for a prolonged period, and Dox was mainly accumulated in tumor tissues (see Supporting Information for detail). Inspired by the promising anticancer efficacy of Dox/siRNA/DNMs *in vitro*, we further evaluated their therapeutic effect in a xenograft tumor model in NOD/SCID mice. When the tumor volume reached approximately 100 mm^3^, the mice were randomly divided into six groups (three mice per group) and given an intratumoral injection of PBS, free siRNA, siRNA/DNMs, free Dox, Dox/DNMs, and Dox/siRNA/DNMs every other day. The dosages of ALK-specific siRNA and Dox were 0.25 mg/kg and 2 mg/kg, respectively, for each injection. **Figure 5A** shows the time schedule of the treatment process performed with eight injections over 22 days. Bodyweight and tumor growth were monitored every other day. No remarkable bodyweight difference and weight loss were observed among the groups except the control group (**Figure 5B**). The relative changes in tumor volumes of the six different treatments are shown in **Figure 5C** and **Figure 5D**. The tumors which were treated with PBS, free siRNA, and siRNA/DNMs grew rapidly and reached ~ 1002, ~ 896, and ~ 784 mm^3^ size after 22 days of treatment. The results demonstrated that siRNA could not provide significant tumor suppression in mice compared with the control group. The tumors treated with free Dox, Dox/DNMs, and Dox/siRNA/DNMs exhibited slower growth than the control group. As expected, the DNMs co-loaded with ALK-specific siRNA and Dox exhibited superior performance in inhibiting tumor growth. The tumor size in Dox/siRNA/DNMs was ~ 231 mm^3^ at the end of treatment. The effective inhibition of tumor growth by Dox/siRNA/DNMs could be attributed to the efficient release and accumulation of Dox and siRNA in tumor tissues, and enhanced chemosensitivity by the ALK-specific siRNA.

**Figure 6:**
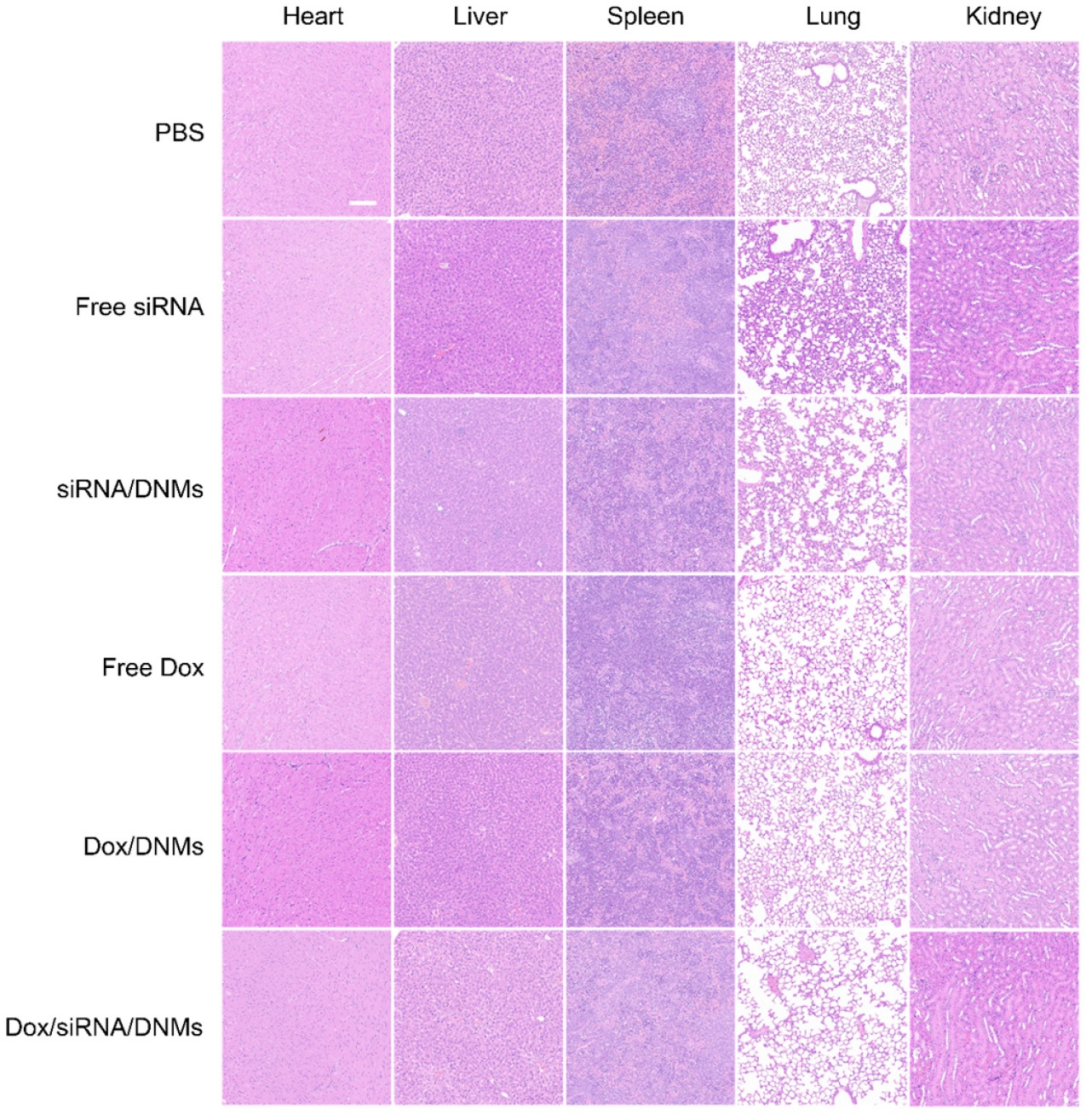
Images of H&E stained tissue slices of the major organs (heart, liver, spleen, lung and kidney) from mice treated with different groups. Scale bar: 100 µm.

The tumor tissues were analyzed with hematoxylin and eosin (H&E) staining (**Figure 5E**). The tissue slices from the Dox/siRNA/DNMs group showed the most significant tumor necrosis and late-stage apoptosis. The terminal deoxynucleotidyl transferase-mediated dUTP nick-end labeling (TUNEL) staining, also demonstrated severe destruction of tumor tissues by Dox/siRNA/DNMs (**Figure 5E**). These results were consistent with the data obtained by H&E staining, confirming the synergistic therapeutic effect of Dox/siRNA/DNMs. The major organs, including the heart, liver, spleen, lung and kidney, were examined by H&E staining to evaluate the toxicity of various treatments (**Figure 6**). Compared to the control group, the tissue slices obtained from the treatment groups showed no apparent morphological difference in H&E staining, demonstrating the low toxic side effects and biosafety of the DNM-based nanocarriers in biomedical applications.

## Experimental

### Materials and Reagents

DL-lactide, 2-azidoethanol and 1, 8-diazabicyclo[5.4.0]undec-7-ene (DBU) were purchased from Shanghai Yuanye Biotechnology Co., Ltd. (Shanghai, China). Cuprous bromide (CuBr) was provided by Macklin Biochemical Co., Ltd. (Shanghai, China). T4 DNA ligase, exonuclease I (Exo I), phi29 DNA polymerase, and 4S Red Plus solution were purchased from BBI Life Sciences Co., Ltd. (Shanghai, China). N,N,N',N'-tetramethylethylenediamine (TEMED) and deoxynucleotides (dNTPs) were obtained from Sangon Biotechnology Co., Ltd. (Shanghai, China). All oligonucleotides used in this study were synthesized and HPLC-purified by Sangon Biotechnology Co., Ltd. (Shanghai, China), and the sequences are listed in **Table S1**. The doxorubicin hydrochloride (Dox·HCl) was ordered from Aladdin (Shanghai, China). The healthy human serum was obtained from the affiliated hospital of Qingdao University (Qingdao, China), and diluted 100 times with ultrapure water for further use. Fetal bovine serum (FBS) and RPMI-1640 medium were purchased from Biological Industries (Israel) and HyClone (USA), respectively. The 1× TAE buffer (40 mM Tris, 1 M glacial acetic acid, 2 mM EDTA-2Na, 15 mM MgCl_2_) with different pH values were used in the experiments. All regents were of analytical grade and used without further purification. Ultrapure water was used during all the experiments.

### Preparation of Primer-PLA Conjugates

The azido-PLA was synthesized via the controlled ring-opening polymerization of DL-lactide 2. In brief, DL-lactide (500.0 mg, 12 eq.) and 2-azidoethanol (25.1 mg, 1 eq.) were dissolved in 5.0 mL of dichloromethane. When the mixture was heated to 40 °C, DBU (66.0 mg, 1.5 eq.) was added, followed by stirring for 30 min. The prepared azido-PLA was characterized by ^1^H NMR. Further, 10 μL of azido-PLA (100 µM), 10 μL of alkyne-modified primer (100 µM) and 40 μL of CuBr (50 μM) were added into 40 µL of 1× TAE buffer at 37 °C for 3 h to form the amphiphilic primer-PLA conjugates via CuAAC reaction.

### Preparation of Dox/siRNA/DNMs

First, 50 μL of 5'-phosphorylated padlock probe (10 μM) was mixed with 100 μL of primer-PLA micelles (10 μM) in 1× T4 ligase buffer (40 mM Tris-HCl, 10 mM MgCl_2_, 10 mM DTT, 0.5 mM ATP, pH 7.8). The mixture was heated at 95 °C for 5 min and gradually cooling to 25 °C at 0.1 °C/s for 3 h to obtain the annealed product of padlock probe and primer-PLA. Then, 10 μL of T4 DNA ligase (5 U/μL) was added to the annealed product to perform the ligation at 16 °C overnight. After that, the reaction temperature was increased to 65 ^o^C for 10 min to inactivate the T4 DNA ligase. Subsequently, the resulting mixture was treated with 2 μL of exonuclease I (Exo I, 20 U/μL) in 1× Exo I buffer (67 mM glycine-KOH, 6.7 mM MgCl_2_, 1 mM DTT, pH 9.5) at 37 °C for 2 h to remove excess primers, followed by heating at 80 °C for 15 min to inactivate Exo I. RCA reaction was carried out by mixing 20 μL of the prepared circular template (0.55 μM), 2 μL of phi 29 DNA polymerase (0.4 U/μL) and 10 μL of dNTPs (2 mM) at 30 °C for 3 h and terminated by heating at 65 °C for 10 min. Then, 2 µL of T1 (0.5 μM), 2 µL of T2 (0.5 μM) and 2 µL of siRNA (0.5 μM) hybridized at 37 °C for 2 h to form the T1-siRNA-T2 hybrids. Subsequently, 12 µL of the RCA products was mixed with the T1-siRNA-T2 hybrids in 12 µL of 1× TAE buffer and reacted at 37 °C for 3 h to obtain the siRNA/DNMs. For Dox intercalation, 3 μL of Dox (83.3 μM) was incubated with the siRNA/DNMs at 37 °C overnight. The final volume was supplemented to 50 μL with 1× TAE buffer (pH 7.4). The fluorescence intensities of Dox were measured on a F-7000 spectrometer (Hitachi, Japan) with the excitation wavelength at 480 nm.

### Native Polyacrylamide Gel Electrophoresis (PAGE)

The 8% acrylamide gel was prepared by mixing 2.7 mL of 30% acrylamide/bis-acrylamide gel solution (29:1), 6.2 mL of ultrapure water, 1 mL of 10× TAE buffer, 90 μL of 10% ammonium persulfate (APS), and 10 μL of N,N,N',N'-tetramethylethylenediamine (TEMED). After polymerization for 30 min at room temperature, the gel was soaked in 1× TAE buffer (pH 8.0). Subsequently, 10 µL of each sample was mixed with 2 μL of 10× loading buffer, and the mixture was added into the resulting 8% native polyacrylamide gel for electrophoresis. The PAGE was running at the voltage of 170 V for 5 min and 110 V for 40 min. The gel was then stained with the diluted 4S Red Plus solution (Sangon Biotech, Shanghai, China) at room temperature for 40 min. Finally, the images of the stained gel were recorded using Tanon 2500R gel imaging system (Tanon Science & Technology Co., Ltd., China).

### Atomic Force Microscopy (AFM) Imaging

A 10 µL of each sample was deposited onto the surface of freshly cleaved mica for 30 min. Then, the mica surface was washed with 30 µL of ultrapure water for three times. After drying in the ambience air, the resultant samples were scanned under the tapping mode using Being Nano-Instruments CSPM-4000 system (Benyuan, China), and the images were analyzed with CSPM Console software (Benyuan-CSPM4000, China).

### Confocal Laser Scanning Microscopy (CLSM)

First, the Karpas 299 (K299) cells were obtained from CoBioer Biosciences Co., Ltd. (Nanjing, China) and cultured in RPMI 1640 medium with 10% (v/v) FBS and 1% penicillin-streptomycin at 37 ^o^C in a humid atmosphere with 5% CO_2_. The cells density was determined using a hemocytometer before each experiment. Then, K299 cells were seeded on the 48-well culture plate (3 × 10^4^ cells per well) and incubated for 12 h. The transfection was performed according to the Xfect^TM^ RNA Transfection Reagent instructions (TaKaRa Biomedical Technology Co., Ltd., China). After transfection for 8 h, the cell nucleus was stained with 10 μL of Hoechst 33342 (Shanghai Sangon Biotech, China) at 37 °C for 30 min. The cells were washed twice with 500 µL of 1× PBS by centrifugation at 1000 rpm for 3 min, followed by resuspending with 300 μL of 1× PBS. Finally, the images were recorded and analyzed by Nikon Confocal Microscope A1 (Nikon, Japan).

### Flow Cytometry Assay

The K299 cells were seeded in 24-well culture plate (5 × 10^4^ cells per well) and cultured in the medium for 12 h. After incubation with FAM-labelled Dox/siRNA/DNMs at 37 ^o^C for 8 h, the cells were washed twice with 500 µL of 1× PBS by centrifugation at 1000 rpm for 3 min, followed by resuspending with 1 mL of 1× PBS in test tubes. The fluorescence signal of 10^4^ cells were determined under 488 nm excitation using Cytomics FC 500 (Beckman, USA).

### Cell Viability Assay

The cell viability was assessed using the CCK-8 assay. The K299 cells were seeded in a 96-well culture plate (10^4^ cells per well) and treated with free siRNA, siRNA/DNMs, free Dox, Dox/DNMs and Dox/siRNA/DNMs, respectively. After treatment for 24 h, 10 μL of CCK-8 solution was added to each well and incubated at 37 ^o^C for another 30 min. The absorbance (A) of each well was measured at 450 nm using a microplate reader (TECAN Safire 2, Switzerland). The percentage of the viable cells was calculated according to the formula of (A_treated_ - A_blank_) /(A_control_ - A_blank_) × 100%.

### *In Vivo* Assays

All animal works were performed in agreement with the Institutional Animal Care and Use Committee. The 4-5 weeks old female NOD/SCID mice weighed between 14-16 g were purchased from Beijing Vital River Laboratory Animal Technology Co., Ltd. (Beijing, China). The tumor xenograft models were established by injecting 6 × 10^6^ K299 cells suspended in 100 μL of PBS into the buttock of the mice. When the tumor grew to 100 mm^3^, the mice were randomly divided into six groups (three mice per group) for different treatment: PBS (control group), free siRNA, siRNA/DNMs, free Dox, Dox/DNMs and Dox/siRNA/DNMs. The mice were treated with equivalent doses (ALK-specific siRNA: 0.25 mg/kg, Dox: 2 mg/kg) via intratumoral injection in a 120 μL of injection volume every three days for eight treatments. Bodyweight and tumor growth were monitored every other day. The length (L) and width (W) of each tumor were recorded and the tumor volumes were calculated with the formula of V = L × W^2^/2. At the end of treatments, the mice were sacrificed, then the tumors were harvested for hematoxylin-eosin (H&E) and TUNEL staining. Besides, the major organs including heart, liver, spleen, kidney and lung from mice in different groups were also harvested for H&E staining. The images were obtained with Pannoramic MIDI (3D HISTECH Ltd., Hungary).

### Statistical Analysis

The results were presented as the mean ± standard deviation (SD). The statistical significance of the differences was measured by a one-way ANOVA (* means p ˂ 0.05, ** means p ˂ 0.01, and *** means p ˂ 0.001).

## Conclusion

We have developed multifunctional pH-responsive DNMs based on RCA technology for the co-delivery of the anticancer drug Dox and therapeutic gene ALK-specific siRNA, achieving a combination of chemotherapy and gene therapy for ALCL both *in vitro* and *in vivo*. The DNA nanovehicles demonstrated several significant advantages for biomedical applications. (1) Easy preparation: the amphiphilic primer-PLA conjugates could be easily synthesized via click chemistry and then self-assembled into micelles. The RCA reaction was performed in situ on the primer-PLA micelles, and the pH-responsive DNMs were readily obtained by crosslinking the RCA products with the triple/helix molecular switch-based probes containing the siRNA. (2) Multifunctionality: the DNMs could be modified in a variety of ways with fluorophores, pH-sensitive domains, drug loading sites, and siRNAs with high payload for bioimaging and acidic tumor microenvironment-responsive drug delivery and gene therapy. Because of the flexible design of the multifunctional DNMs, the therapeutic components could be readily incorporated into the DNMs, to extend their capabilities and applications. (3) Enhanced therapeutic efficiency: the DNMs with good biostability and biocompatibility could protect Dox and siRNA from random release and degradation in the extracellular environment, resulting in a significant chemotherapeutic effect and gene silencing efficiency in K299 cells. Thus, the chemotherapeutic drug and specific siRNA synergistically enhanced cell apoptosis and repressed tumor growth in a mice xenograft model. In summary, the pH-responsive DNMs achieved co-delivery of anticancer drugs and therapeutic genes, which holds great promise for the development of nanotheranostic platforms for synergetic cancer therapy.

## Supplementary Material

Supplementary methods, figures and tables.

## Abbreviations

DNMs: DNA nanomicelles
Dox: doxorubicin
NPM-ALK: nucleophosmin-anaplastic lymphoma kinase
ALCL: anaplastic large cell lymphoma
RCA: rolling circle amplification
PLA: polylactide
RNAi: RNA interference
RISC: RNA induced silencing complex
ssDNA: single-stranded DNA
dNTPs: deoxynucleotide triphosphates
CuAAC: copper (I)-catalyzed alkyne-azide cycloaddition
PAGE: polyacrylamide gel electrophoresis
CMC: critical micelle concentration
Exo I: exonuclease I
AFM: atomic force microscopy
CLSM: confocal laser scanning microscopy
qRT-PCR: quantitative real-time polymerase chain reaction
CI: combination index
H&E: hematoxylin and eosin
TUNEL: terminal deoxynucleotidyl transferase-mediated dUTP nick-end labeling
