# Supplementary material for: pH-responsive DNA nanomicelles for chemo-gene synergetic therapy of anaplastic large cell lymphoma
Source: Theranostics. 2020 Jul 9;10(18):8250–63. doi: 10.7150/thno.45803 (PMC7381733; doi:10.7150/thno.45803)
Supplement: Supplementary file 1 — Supplementary methods, figures and tables. [file thnov10p8250s1.pdf]

Supplementary Material

pH-responsive DNA nanomicelles for chemo-gene synergetic therapy of anaplastic large cell lymphoma

Yuwei Li[1†], Shuzhen Yue[1†], Yixiu Wang[3], Chengzhan Zhu[3], Jingyu Cao[3], Weiling Song[2], Sai Bi[1, ✉]

1. Research Center for Intelligent and Wearable Technology, College of Chemistry and Chemical Engineering, Qingdao University, Qingdao 266071, P. R. China.
2. Laboratory of Optic-electric Sensing and Analytical Chemistry for Life Science, MOE, Shandong Key Laboratory of Biochemical Analysis, Key Laboratory of Analytical Chemistry for Life Science in Universities of Shandong, College of Chemistry and Molecular Engineering, Qingdao University of Science and Technology, Qingdao 266042, P. R. China
3. Department of Hepatobiliary and Pancreatic Surgery, Affiliated Hospital of Qingdao University, Qingdao 266003, P. R. China

✉ Corresponding Author. E-mail address: bisai11@126.com.

† These authors contributed equally to this work.

S1. Additional experimental section

S1.1. Quantitative Real-Time PCR (qRT-PCR) Analysis

The K299 cells were seeded in 24-well culture plate (5×10^4 cells per well). After culturing in the medium overnight, the cells were transfected with free siRNA, siRNA/DNMs and negative control (NC) siRNA/DNMs for 48 h at 37 ^oC, respectivly. Then, the cells were incubated with 500 μL RNAiso plus reagent on ice for 45 min to obtain lysate. Next, the lysate was added with 100 μL of chloroform to incubate at room temperature for 5 min, followed by centrifugation at 12,000 rpm for 15 min at 4 ^oC to separate the aqueous and organic phase. Subsequently, 200 μL of isopropanol was added into the obtained aqueous phase to precipitate the RNA. After incubation for 10 min, the mixture was centrifuged at 12,000 rpm for 15 min at 4 ^oC to discard the supernatant. The precipitation was washed with 200 μL of ethanol via centrifugation at 12,000 rpm for 10 min at 4 ^oC. Finally, the obtained RNA was dissolved in 20 μL of DEPC-treated water and the concentration was measured with Micro Nucleic Acid Protein Analyzer (USA). The mRNA in total RNA was reversely transcribed into cDNA by PrimeScript™ RT reagent kit (TaKaRa, China) according to the indicated protocol. The quantitative real-time PCR analysis was carried out with TB Green® Premix Ex Taq™ II (TaKaRa, China) on a Roche LightCycler® 480 II (Switzerland). The reaction solution contained 5 μL of DEPC-treated water, 10 μL of TB Green, 1 μL of cDNA, 2 μL of forward primer and 2 μL of reverse primer (The sequences of the PCR primers are listed in **Table S1**). The conditions of PCR contained an initial denaturation step at 95 ^oC for 30 s, followed by 40 cycles each of 95 ^oC for 5 s and 60 ^oC for 30 s. The data of mRNA expression was evaluated by normalizing to the expression of β-actin and using $2^{-\Delta\Delta Ct}$ method.

S1.2. Western Blotting

The K299 cells were seeded in 6-well culture plate (1×10^5 cells per well). After culturing in the medium overnight, the cells were transfected with free siRNA, siRNA/DNMs and NC siRNA/DNMs for 48 h at 37 ^oC, respectivly. The cells were lysed via RIPA buffer containing protease inhibitor (RIPA: PIC: PMSF = 100: 1: 1) on ice. Then, the lysate was centrifuged at

13,000 rpm for 15 min at 4 °C and the supernatant proteins were transferred to a fresh centrifuge tube. The concentrations of each sample were measured using BCA protein assay kit (CoWin Biosciences, Beijing, China). The extracted proteins were separated with 10% SDS-PAGE at the voltage of 80 V for 30 min and 120 V for 45 min. Then the bands were transferred to the polyvinylidene fluoride (PVDF) membrane on the ice water bath with the current of 290 mA for 1.5 h, followed by incubating in blocking solution containing 5% skim milk for 1 h at room temperature. After washing three times with 1× PBST (1× PBS: Tween 20 = 1000: 1), the membrane were incubated with ALK (31F12) mouse mAb and GAPDH (D16H11) XP® rabbit mAb (Cell Signaling Technology, Inc., USA) at 4 °C overnight, respectively, and subsequently incubated with the corresponding secondary antibodies (horseradish peroxidase (HRP)-linked anti-mouse and anti-rabbit IgG, Cell Signaling Technology, Inc., USA) at room temperature for 1 h. The detection was performed with Sparkjade ECL super (Sparkjade Biotechnology, Shandong, China) using Vilber Fusion FX7 Spectra (France).

S1.3. Apoptosis Assays by AnnexinV-APC/7-AAD Staining

The K299 cells were seeded in 24-well culture plate (5×10^4 cells per well). After culturing in the medium for 12 h, the cells were treated with free siRNA, siRNA/DNMs, free Dox, Dox/DNMs, and Dox/siRNA/DNMs for 48 h, respectively. Then the cells were collected and washed with PBS twice via centrifugation at 1000 rpm for 5 min, followed by staining with Annexin V-APC/7-AAD apoptosis kit (Multi Sciences (LIANKE) Biotech, Co., Ltd., Hangzhou, China) according to the instructions. The results were analyzed using Cytomics FC 500 (Beckman, USA) by counting 10^4 events.

S1.4. Calcein AM/PI Assay

The K299 cells were seeded in 24-well culture plate (5×10^4 cells per well) and cultured in the medium for 12 h. The cells were transfected with free siRNA, siRNA/DNMs, free Dox, Dox/DNMs, and Dox/siRNA/DNMs for 48 h, respectively. Then, the cells were collected and stained with Calcein-AM/PI double stain kit (Yeasen Biotech Co., Ltd., Shanghai) according

to the instructions. The live/dead images were recorded on Nikon Confocal Microscope A1 (Nikon, Japan).

S1.5. Pharmacokinetics Analysis

For pharmacokinetics studies, female NOD/SCID mice weighing between 14-16 g were purchased and divided into two groups. The mice were treated with free Dox and Dox/siRNA/DNMs at a dose of 2 mg/kg Dox (3 mice for each group), respectively. After injection, 500 μL of blood samples were collected into the heparin-treated tubes at different time points, followed by centrifugation at 5,000 rpm for 5 min at room temperature to obtain plasma. 100 μL of the resulting plasma was added to 100 μL of deionized water and 1 mL of ethanol, followed by adding 25 μL of 20% sodium dodecyl sulfate (SDS). Subsequently, the samples were vortexed for 30 s and centrifuged at 12,000 rpm for 10 min at 4 ^{o}C to extract Dox. The concentration of Dox in plasma was measured by F-7000 spectrometer (Hitachi, Japan) and calculated via standard curve[1].

Table S1. Oligonucleotides sequences used in this work.

Name	Sequences (5′- 3′)
primer	CH≡C-TTTTTTTTTTTTACTGGGCGAAACAAGTCTATTGACTATGAGC
padlock probe	Phosphate-CTTGTTTCGCCCAGTGGAATAATGACAATCATAATGAGCATAGGTAT TTCGCGCTCATAGTCAATAGA
T1	GGAGGGAGGGAGGTTTACCTCCCTCCCTCCCTTTGCCTCCCTCCCTCCAGCAT AATGAGAATCATA-FAM
T2	FAM-GAGCATAGGTATTTCCGACCTCCCTCCCTCCGTTTCCCTCCCTCCCTCCA TTTGGAGGGAGGGAGG
anti-ALK siRNA	antisense: GGCGGUACACUACUAAGUGTT<br>sense: GCUGGAGGGACACUUAGUAGUGUACCGCCTTAGGGAGGUCG
forward primer (β-actin)	CCTCTCCCAAGTCCACACAG
NC siRNA	antisense: GGACCACCGCAUCUCUACAUU<br>sense: UGUAGAGAUGCGGUGGUCCUU
forward primer (anti-ALK)	ACAGGCCCAACTTTGCCATC
reverse primer (anti-ALK)	TATCGGCAAAGCGGTGTTGA
forward primer (NC)	GGCATGGGTGCCCCGACGTT
reverse primer (NC)	AGAGGCCTCAATCCATGGCA
forward primer (β-actin)	CCTCTCCCAAGTCCACACAG
reverse primer (β-actin)	GGGCACGAAGGCTCATCATT

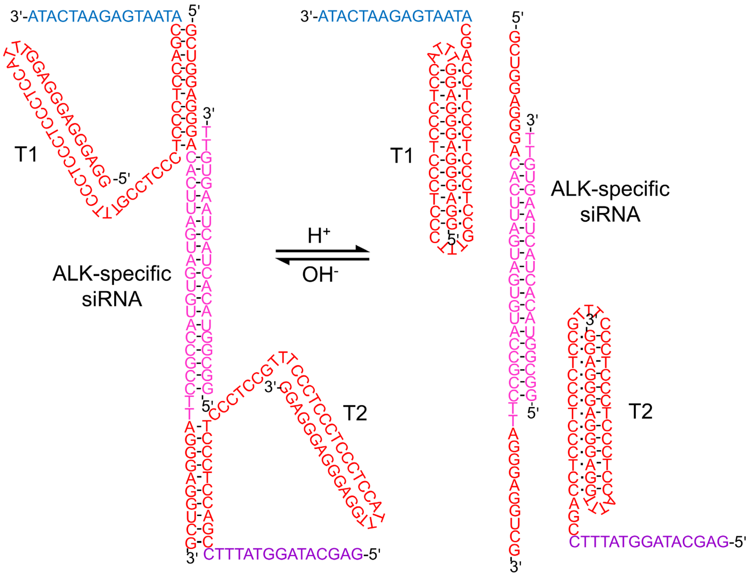

Figure S1. Base composition and secondary structures of T1-siRNA-T2 hybrid in response to pH.

S2. Characterization of Azido-PLA

The ^{1}H NMR spectrum of the synthesized azido-PLA in $CDCl_3$ is shown in **Figure S2A**. ^{1}H NMR (δppm, $CDCl_3$): 5.10-5.16 (1H, b), 4.32-4.34 (2H, c), 3.38-3.53 (2H, d), 1.64-1.65 (3H, a). The molecular formula of N_3-PLA was 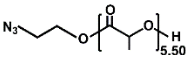 and the molecule weight of azido-PLA was calculated as 483 g/mol. The Fourier transform infrared (FTIR) spectra for azido-PLA sample is depicted in **Figure S2B**. The characteristic absorption peaks of 2991.96 and 2941.38 cm^{-1} were attributed to the asymmetric and symmetric modes of C-H stretching, respectively. Moreover, the absorption peak appeared at 2103.35 cm^{-1} belonged to the azide groups, confirming the successful synthesis of azido-PLA. The strong peak at 1739.98 cm^{-1} belonged to the C=O bond stretch, and the appearance of peak at 1451.38 cm^{-1} was attributed to the CH_3 band. Besides, the peak of 1324.28 cm^{-1} in the spectra indicated the deformation of C-H. The C-O-C asymmetric stretching of ester groups was confirmed by the appearance of peak at 1090.20 cm^{-1}.

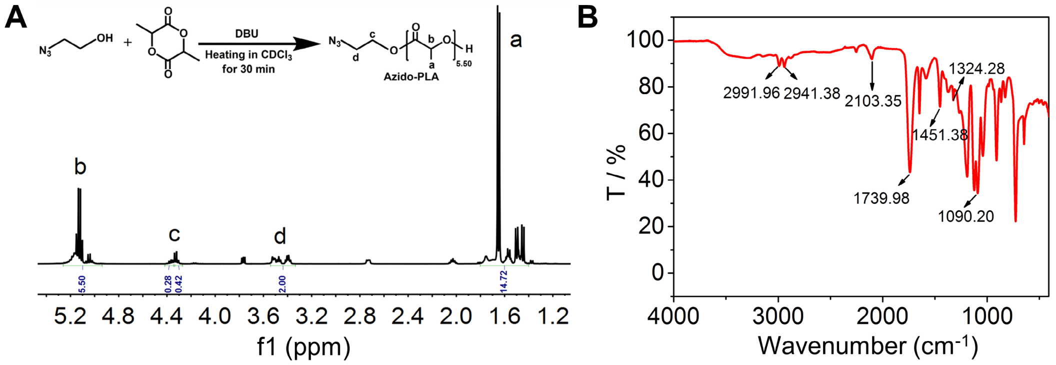

Figure S2. Characterization of azido-PLA. (A) ^{1}H NMR spectra of azido-PLA. Inset: synthesis of azido-PLA. (B) FTIR spectra of azido-PLA.

S3. Optimization of RCA Reaction

The reaction temperature and reaction time of RCA have been optimized as follow. From **Figure S3**, the RCA reaction was performed at 25 °C and 30 °C for 5 h, respectively. When the samples were incubated at 30 °C for 3 h, the concentration of DNMs reached the highest and no obvious increase was observed in the next two hours. Thus, the optimal reaction conditions for RCA were selected as 30 °C for 3 h.

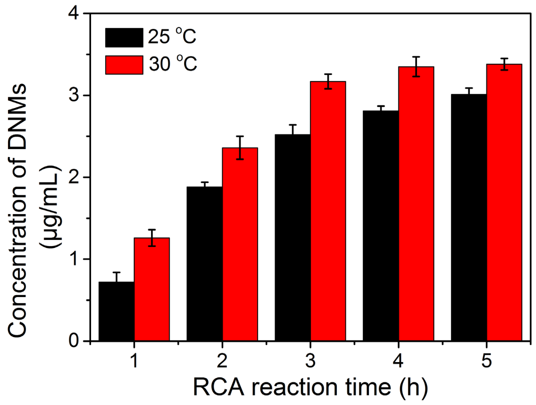

Figure S3. Effects of reaction temperature and reaction time of RCA on the concentration of DNMs. Error bars indicate SD (n = 3).

S4. Scanning Electron Microscopy (SEM) Characterization

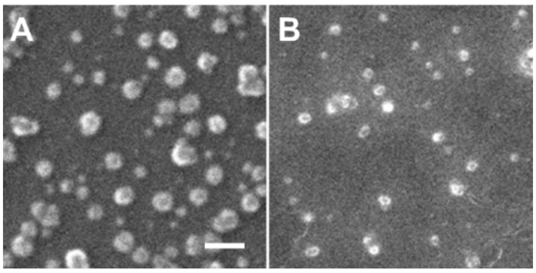

Figure S4. SEM images of DNMs (A) and siRNA/DNMs (B) at pH 7.0. Scale bar: 500 nm.

S5. Dynamic Light Scattering (DLS) Characterization

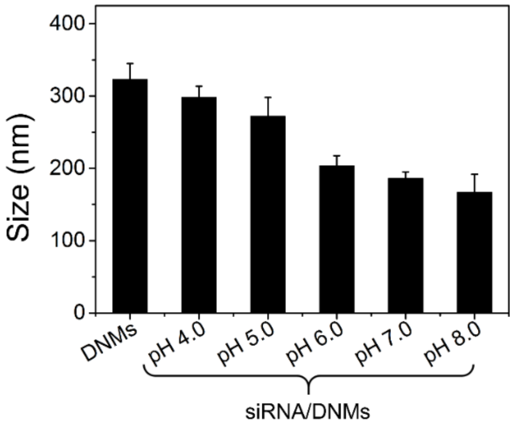

Figure S5. Size distributions of DNMs and siRNA/DNMs at different pH measured by DLS. Error bars indicate SD (n = 3).

S6. Loading efficiency and capacity of siRNA and Dox in DMNs

The loading efficiency and loading capacity of siRNA and Dox are calculated as follows [2].

Loading efficiency and capacity of siRNA. Firstly, 5 µL of T1 (0.5 µM), 5 µL of T2 (0.5 µM) and 5 µL of siRNA (0.5 µM) were mixed and reacted at 37 °C for 2 h to form the T1-siRNA-T2 hybrids. Then, the T1-siRNA-T2 reacted with 24 µL of DNMs at 37 °C for 3 h to obtain the siRNA/DNMs. The mixture was centrifuged at 13,000 rpm for 10 min. The supernatant was collected and supplied to 100 µL with 1× TAE (pH 7.4) and the concentration of siRNA was measured by Micro Nucleic Acid Protein Analyzer. The loading efficiency and capacity of siRNA in DNMs can be calculated as follows:

Mass of added siRNA: $m_{added} = n_{added} \times M_{added} = C_{added} \times V_{added} \times M_{siRNA} = 0.5 \times 10^{-6}$ mol/L × 5 μL × 18780.46 g/mol = 0.047 μg

Concentration of siRNA in the supernatant measured with Micro Nucleic Acid Protein Analyzer: $C_{unloaded} = 0.10$ μg/mL

Mass of unloaded siRNA: $m_{unloaded} = C_{unloaded} \times V = 0.10$ μg/mL × 100 μL × 10^{-3} = 0.010 μg

Mass of loaded siRNA: $m_{loaded} = m_{added} - m_{unloaded} = 0.047$ μg - 0.010 μg = 0.037 μg

Thus, siRNA loading efficiency = $m_{loaded\ siRNA}/m_{added\ siRNA}$ = 0.037/0.047 × 100% = 78.7 %

Concentration of DNMs measured with Micro Nucleic Acid Protein Analyzer: $C_{DNMs} = 3.17$ μg/mL

Mass of DNMs: $m_{DNMs} = C_{DNMs} \times V_{DNMs} = 3.17$ μg/mL × 24 μL × 10^{-3} = 0.076 μg

Thus, siRNA loading capacity = $m_{loaded\ siRNA}/(m_{loaded\ siRNA} + m_{DNMs})$ × 100% = 0.037/(0.037 + 0.076)] × 100% = 32.7 %, and per gram of DNMs can load 0.49 g siRNA (0.037/0.076 = 0.49).

Loading efficiency and capacity of Dox. First, a series of Dox with different concentrations were prepared to obtain the corresponding UV-vis absorbance. The standard curve of UV-vis absorbance versus Dox concentration (1-10 μM) is shown in **Figure S6**. The linear regression equation is expressed as A = 0.0894C + 0.0703 (C is Dox concentration; A is UV-vis absorbance), n = 5, $R^2 = 0.9948$.

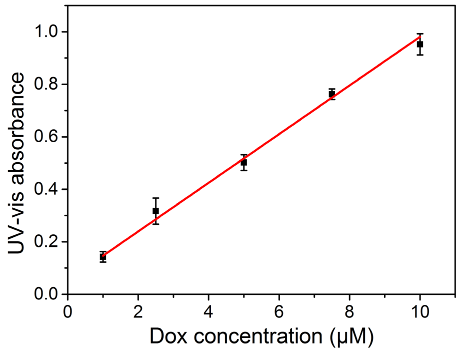

Figure S6. Standard curve of UV-vis absorbance versus Dox concentration.

For the preparation of siRNA/DNMs, 4 μL of T1 (0.5 μM), 4 μL of T2 (0.5 μM) and 4 μL of siRNA (0.5 μM) were mixed and reacted at 37 °C for 2 h to form the T1-siRNA-T2 hybrids, followed by incubation with 24 μL of DNMs at 37 °C for 3 h to obtain the siRNA/DNMs (36 μL in total). Then, 10 μL of 100 μM Dox was added into 36 μL of siRNA/DNMs. After incubation at 37 °C overnight, the mixture was centrifuged at 14,000 rpm for 15 min. The unloaded Dox in the supernatant was collected and supplied to 100 μL with 1× TAE (pH 7.4), followed by measuring the UV-vis absorbance at 480 nm on a BioTek microplate reader.

UV-vis absorbance of unloaded Dox in supernatant: $A_{unloaded} = 0.483$

According to the standard curve (**Figure S6**), the concentration of unloaded Dox in supernatant: $C_{unloaded} = (0.483-0.0703) / 0.0894 = 4.62$ μM

Mole of unloaded Dox: $n_{unloaded} = C_{unloaded} \times V_{unloaded} = 4.62 \times 10^{-6}$ mol/L × 100 μL = 4.62×10^{-4} μmol

Mole of added Dox: $n_{added} = C_{added} \times V_{added} = 10 \times 10^{-6}$ mol/L × 100 μL = 10^{-3} μmol

Mole of loaded Dox: $n_{loaded} = n_{added} - n_{unloaded} = 10^{-3}$ μmol - 4.62×10^{-4} μmol = 5.38×10^{-4} μmol

Thus, Dox loading efficiency = $n_{loaded\ Dox}/n_{added\ Dox} \times 100\% = 53.8\%$

Mass of loaded Dox: $m_{added} = n_{added} \times M_{Dox} = 5.38 \times 10^{-4}$ μmol × 579.98 g/mol = 0.312 μg

Concentration of DNMs measured with Micro Nucleic Acid Protein Analyzer: $C_{DNMs} = 3.17$ μg/mL

Mass of DNMs: $m_{DNMs} = C_{DNMs} \times V$ = 3.17 μg/mL × 24 μL × 10^{-3} = 0.076 μg

Mass of siRNA: $m_{siRNA} = n_{siRNA} \times M_{siRNA} = C_{siRNA} \times V_{siRNA} \times M_{siRNA} = 0.5 \times 10^{-6}$ mol/L × 4 μL × 18780.46 g/mol = 0.038 μg

Mass of T1: $m_{T1} = n_{T1} \times M_{T1} = C_{T1} \times V_{T1} \times M_{T1} = 0.5 \times 10^{-6}$ mol/L × 4 μL × 20137.06 g/mol = 0.040 μg

Mass of T2: $m_{T2} = n_{T2} \times M_{T2} = C_{T2} \times V_{T2} \times M_{T2} = 0.5 \times 10^{-6}$ mol/L × 4 μL × 20146.02 g/mol = 0.040 μg

Mass of siRNA/DNMs: $m_{siRNA/DNMs} = m_{DNMs} + m_{siRNA} + m_{T1} + m_{T2} = 0.076 + 0.038 + 0.040 + 0.040 = 0.194$ μg

Thus, Dox loading capacity = $m_{loaded\ Dox}/(m_{loaded\ Dox} + m_{siRNA/DNMs}) = 0.312/(0.312 + 0.194) \times 100\% = 61.7$ %, and per gram of siRNA/DNMs can load 1.6 g Dox (0.312/0.194 = 1.6).

S7. Stability of siRNA/DNMs

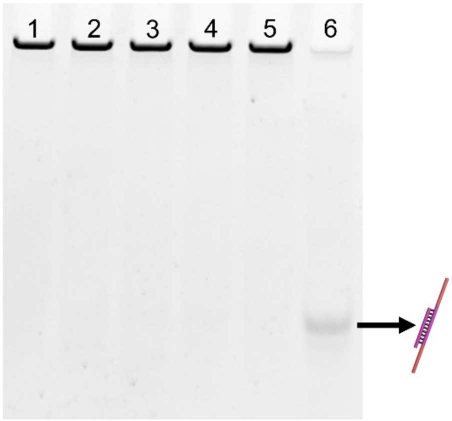

Figure S7. Stability of siRNA/DNMs incubated in healthy human serum for different time. Lane 1: 2 h; Lane 2: 4 h; Lane 3: 6 h; Lane 4: 8 h; Lane 5: 10 h; Lane 6: siRNA.

S8. Knockdown Efficiency of Negative Control siRNA/DNMs

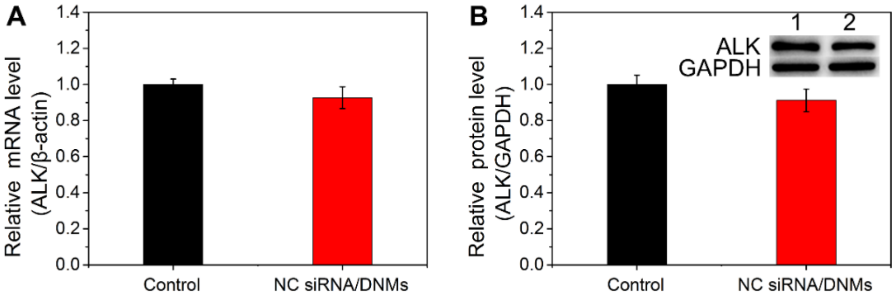

Figure S8. The mRNA (A) and protein (B) expression levels of ALK gene in K299 cells without treatment (control) and treated with NC siRNA/DNMs. Inset: The corresponding western blotting analysis. Lane 1: control; lane 2: NC siRNA/DNMs. Error bars indicate SD (n = 3).

S9. Combination Index (CI) Calculation

To confirm the synergistic therapy effect of Dox/siRNA/DNMs, the combination index (CI) was calculated using the formula as follows: $CI = C_1/C_{x1} + C_2/C_{x2}$, in which C_1 is the concentration of drug 1 required to achieve a certain effect in the combination, C_{x1} is the concentration of drug 1 that causes an identical effect alone; C_2 is the concentration of drug 2 required to achieve a certain effect in the combination, C_{x2} is the concentration of drug 2 that generates an identical effect alone. CI > 1 indicates antagonism effect, CI = 1 indicates an additive effect, and CI < 1 indicates synergistic effect [3]. In this work, IC50 values (half maximal inhibitory concentration) of siRNA/DNMs, Dox/DNMs and Dox/siRNA/DNMs were used to calculate CI. IC50 of siRNA/DNMs and Dox/DNMs in K299 cells was 6.87 μg/mL and 16.85 μg/mL, respectively. Besides, when the survival rate reached to 50%, the concentrations of siRNA and Dox in Dox/siRNA/DNMs were 2.13 and 8.76 μg/mL, respectively. Therefore, the CI of siRNA and Dox delivered via DNMs was 2.13/6.87 + 8.76/16.85 = 0.83 < 1, indicating the synergistic effect.

S10. Pharmacokinetics Analysis

Pharmacokinetic curves of Dox and relevant pharmacokinetic parameters are shown in **Figure S9** and **Table S2**, respectively. For the Dox/siRNA/DNMs treated group, the area under the curve ($AUC_{0-\infty}$) was over 10-fold higher than that of the free Dox treated group (325.92 ± 3.62 μg/mL×h vs 32.03 ± 1.35 μg/mL×h). Meanwhile, the half-time of Dox in Dox/siRNA/DNMs increased to 3.61 ± 1.12 h. All the results demonstrated that the Dox/siRNA/DNMs were able to maintain a high concentration of Dox in the prolonged period of systemic circulation.

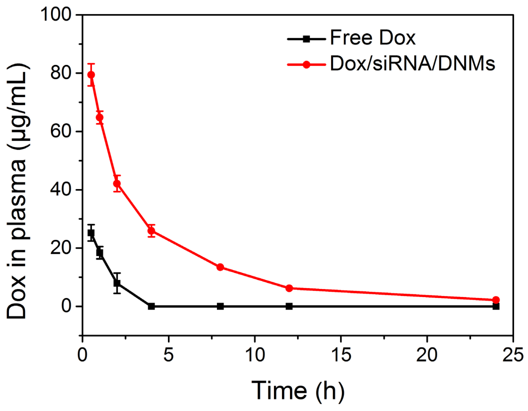

Figure S9. Pharmacokinetic curves of Dox after injection of free Dox and Dox/siRNA/DNMs in mouse plasma, respectively. Error bars indicate SD (n = 3).

Table S2. Pharmacokinetic parameters of free Dox and Dox/siRNA/DNMs.

Parameter	Free Dox	Dox/siRNA/DNMs
$AUC_{0\text{-}\infty}$ (μg/mL×h)	32.03 ± 1.35	325.92 ± 3.62
C_{max} (μg/mL)	25.24 ± 2.8	79.41 ± 3.8
$t_{1/2}$ (h)	1.82 ± 0.06	3.61 ± 1.12

AUC: area under curve; C_{max}: maximum plasma drug concentration; $t_{1/2}$: plasma half-life

S11. Biodistribution Assay

The biodistribution assay was carried out via intratumor injection, in which the distribution of Dox in tumor tissues and other organs (heart, liver, spleen, lung and kidney) was measeured in NOD/SCID mice after 4 h injection. As shown in the following **Figure S10**, Dox was mainly distributed in the tumor site and slightly in kidney and liver. Notably, Dox/siRNA/DNMs-treated group exhibited the higher Dox accumulation in the tumor tissues compared to the free Dox-treated group, which could be attributed to the enhanced permeability and retention (EPR) effects [4]. The above results demonstrated that the Dox/siRNA/DNMs could efficiently deliver Dox to the tumor tissues for effective cancer therapy.

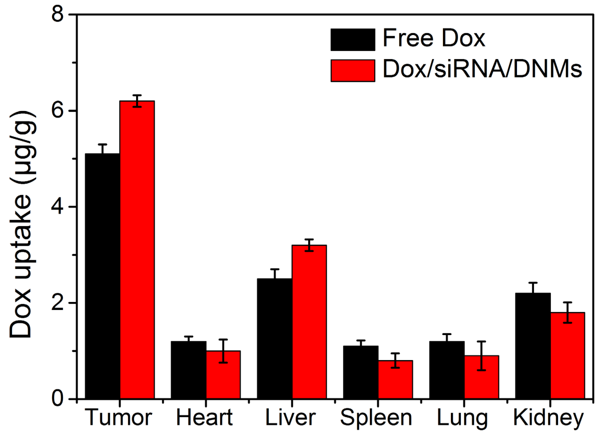

Figure S10. Quantitative analysis of Dox distribution in tumor and organs of NOD/SCID mice. Error bars indicate SD (n = 3).

References

1. Yang T, Li B, Qi S, Liu Y, Gai Y, Ye P, et al. Co-delivery of doxorubicin and Bmi1 siRNA by folate receptor targeted liposomes exhibits enhanced anti-tumor effects in vitro and in vivo. Theranostics. 2014; 4: 1096-111.

2. Zhang L, Abdullah R, Hu X, Bai H, Fan H, He L, et al. Engineering of bioinspired, size-controllable, self-degradable cancer-targeting DNA nanoflowers via the incorporation of an artificial sandwich base. J Am Chem Soc. 2019; 141: 4282-90.

3. Li P, Yang S, Dou M, Chen Y, Zhang J, Zhao X. Synergic effects of artemisinin and resveratrol in cancer cells. J Cancer Res Clin Oncol. 2014; 140: 2065-75.

4. He X, Hai L, Su J, Wang K, Wu X. One-pot synthesis of sustained-released doxorubicin silica nanoparticles for aptamer targeted delivery to tumor cells. Nanoscale. 2011; 3: 2936-42.
